# Supplementary material for: Ultra-Low Thermal Conductivity of Moiré Diamanes
Source: Membranes (Basel). 2022 Sep 25;12(10):925. doi: 10.3390/membranes12100925 (PMC9607344; doi:10.3390/membranes12100925)
Supplement: Supplementary file 1 [file membranes-12-00925-s001.zip › membranes-1910833-supplementary.pdf]

Supplementary Materials: Ultra-Low Thermal Conductivity of Moiré Diamanes

Suman Chowdhury [1,*], Victor A. Demin [2], Leonid A. Chernozatonskii[2,*] and Alexander G. Kvashnin[3,*]

1. Structure

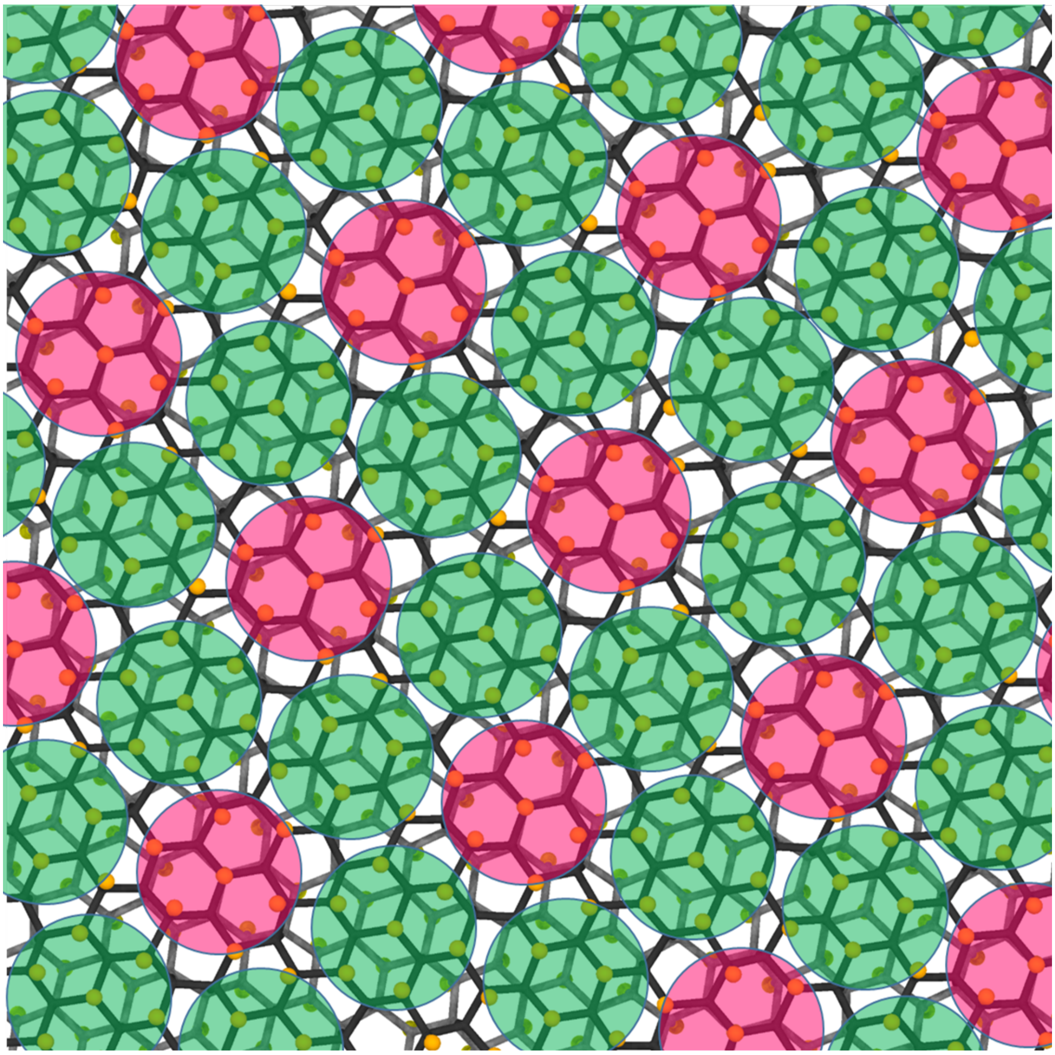

Figure S1. Highlighted by red and green color domains of AA- and AB-stacked diamanes, respectively, inside the *Dn*13 Moiré structure

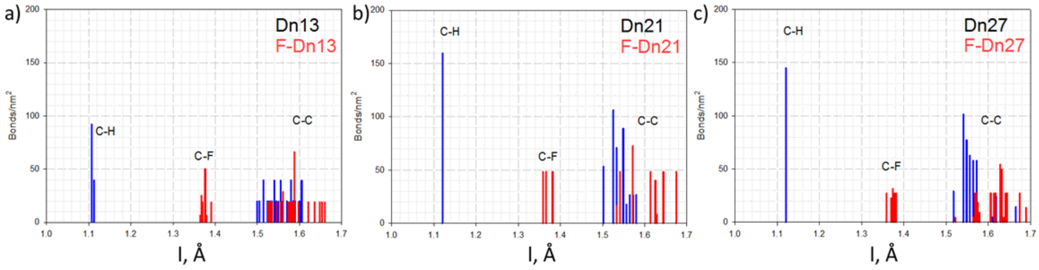

Figure S2. Distributions of bond lengths in considered Moiré diamanes

2. Lattice Thermal Conductivity as Function of Temperature

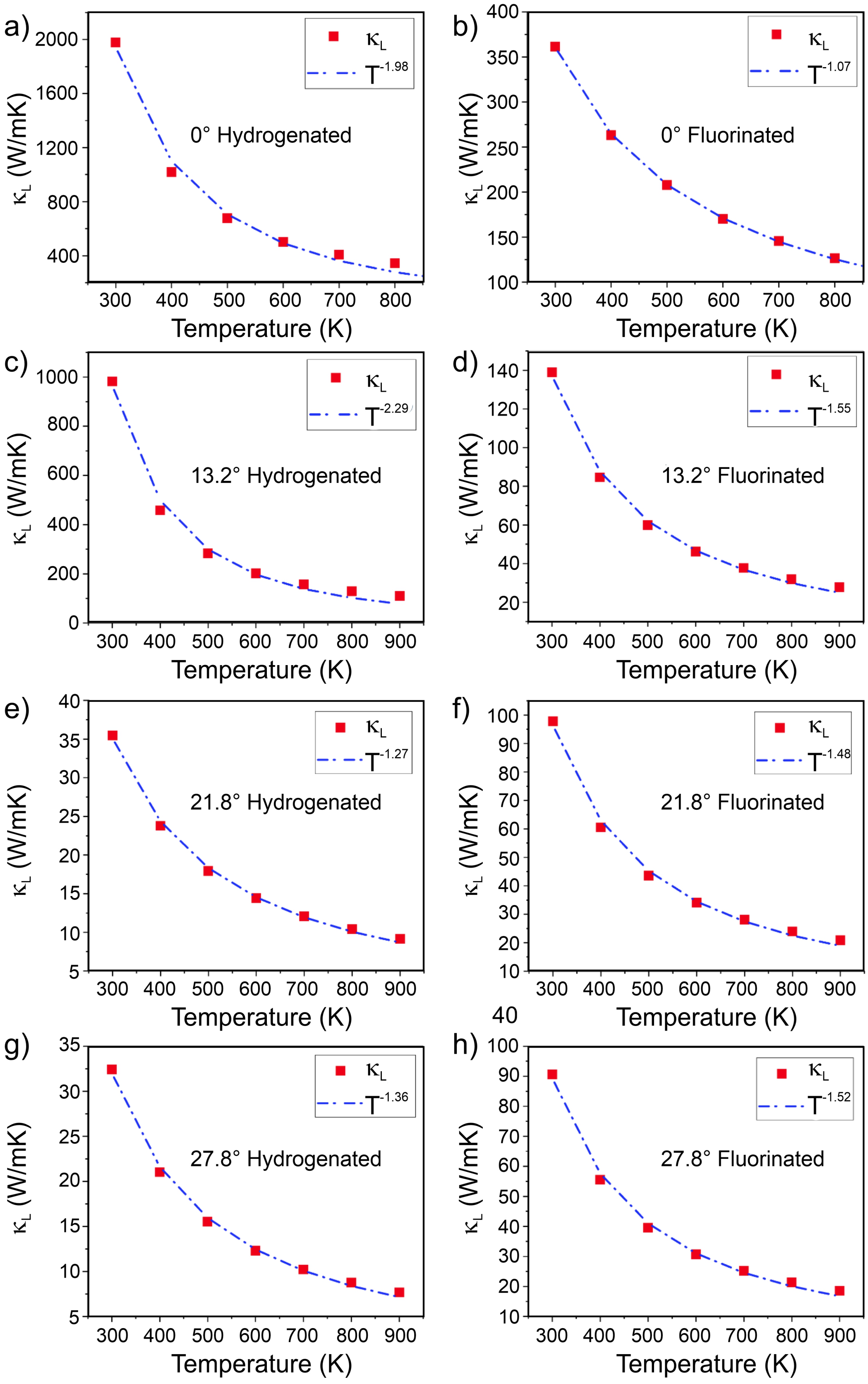

Figure S3. Fitted dependencies of lattice thermal conductivity on temperature for studied Moiré diamanes with different twist angles of (a, b) 0°, (c,d) 13.2°, (e, f) 21.8° and (g, h) 27.8°

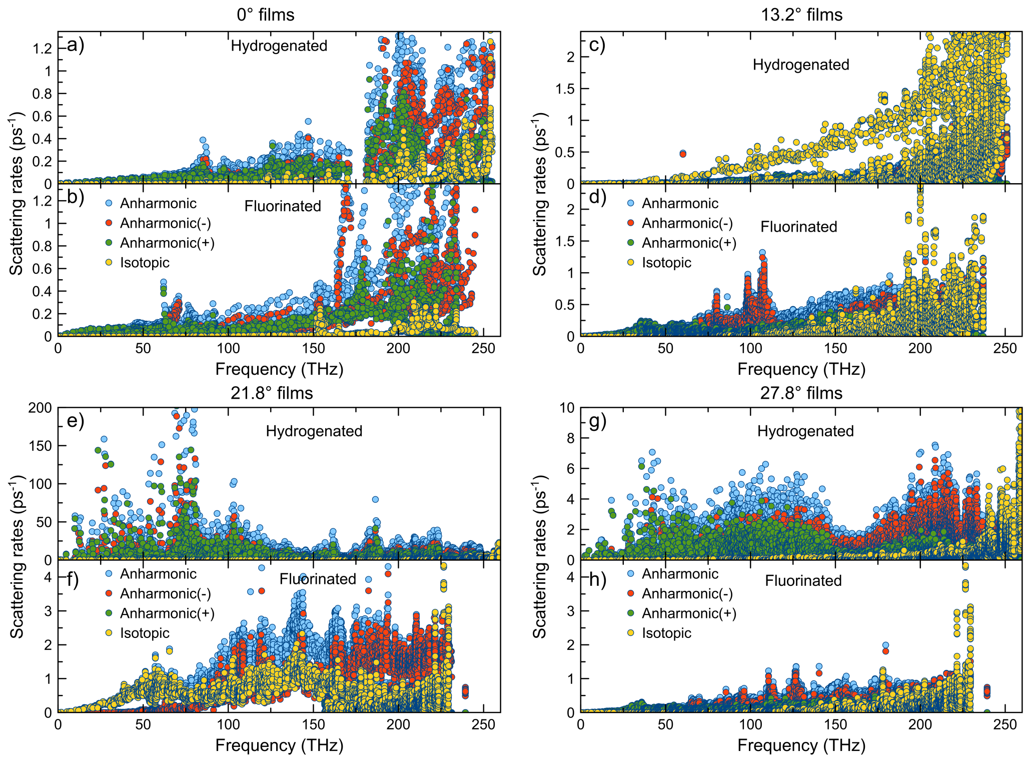

Figure S4. Three-phonon scattering rates for considered hydrogenated and fluorinated Moiré diamanes with twist angles of (a, b) 0°, (c, d) 13.2°, (e, f) 21.8° and (g, h) 27.8°.

3. *Grüneisen* Parameter

From the Figures S5 and S6, we can clearly see that the value of the γ parameter of the hydrogenated films with twist angles 21.8° and 27.8° are more than that of corresponding fluorinated films. This proves that hydrogenated films are more anharmonic in comparison with their fluorinated counterpart. It can even also be seen that the hydrogenated films become more anharmonic with increasing twist angle. Now, in all the twisted systems, negative values of γ can be observed, which is a very common phenomenon that can also be noticed in other 2D materials [3]. The *Grüneisen* parameter is related to linear thermal expansion. Thus, its negative value indicates negative thermal expansion of the twisted systems. The *Grüneisen* parameter is important in the sense that it can be a technological key to suitably control thermal expansion over a range of frequencies and temperatures. From that point of view, we can comment that twisted bi-layer hydrogenated or fluorinated systems may indicate a new direction in the field of negative thermal expansion [1–3].

1. Ghosal, S.; Chowdhury, S.; Jana, D. Electronic and Thermal Transport in Novel Carbon-based Bilayer with Tetragonal Rings: A Combined Study using First-principles and Machine Learning Approach. *Phys. Chem. Chem. Phys.* **2021**, *23*, 14608–14616.
2. Takenaka, K. Negative thermal expansion materials: technological key for control of thermal expansion. *Sci. Technol. Adv. Mater.* **2012**, *13*, 013001.
3. Sanson, A.; Chen, J. Towards the control of thermal expansion: From 1996 to today *Front. Chem.* **2019**, *7*, 284.

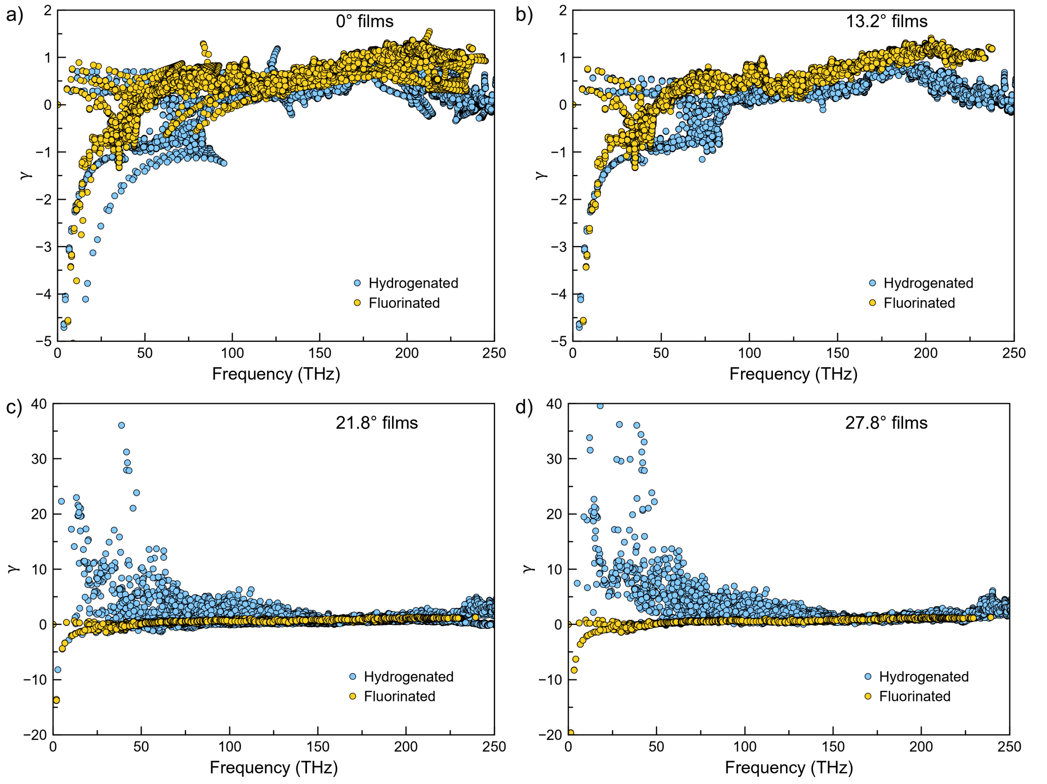

Figure S5. Phonon frequency dependent *Grüneisen* parameter γ of Moiré diamane films with a) 0°, b) 13.2° c) 21.8°, and d) 27.8° twist angles

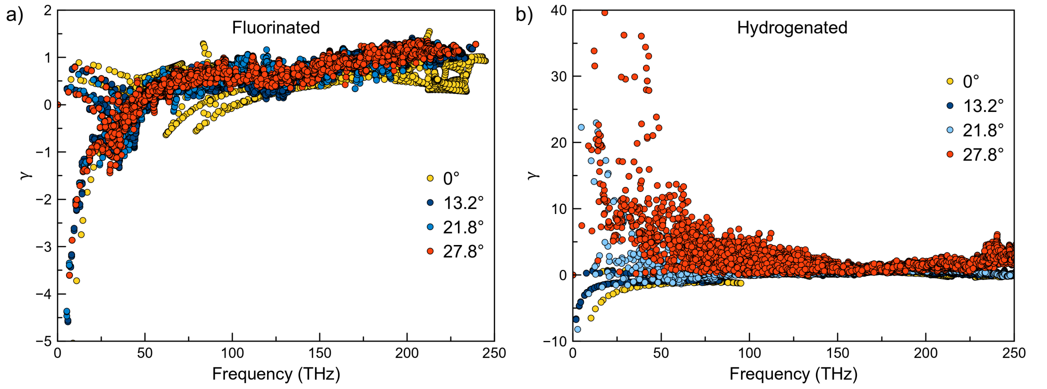

Figure S6. Phonon frequency dependent *Grüneisen* parameter γ for a) fluorinated and b) hydrogenated Moiré diamanes with different twist angles

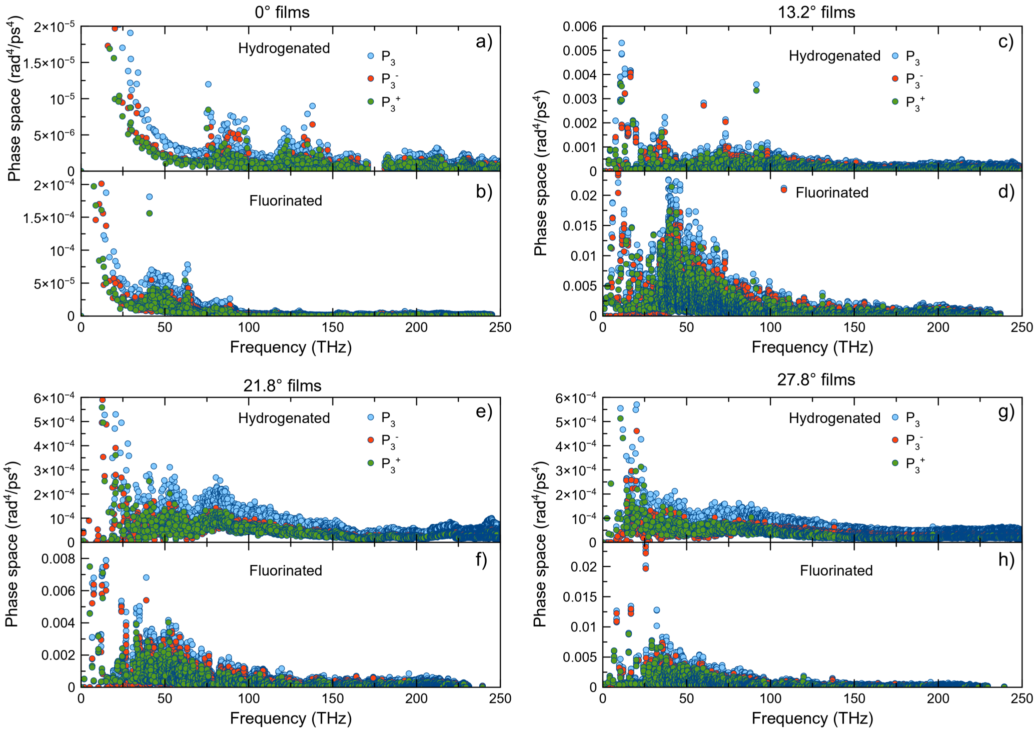

Figure S7. Three-phonon scattering phase space for considered films with twist angles of a, b) 0°, c,d) 13.2° e, d) 21.8°, and g, h) 27.8°.

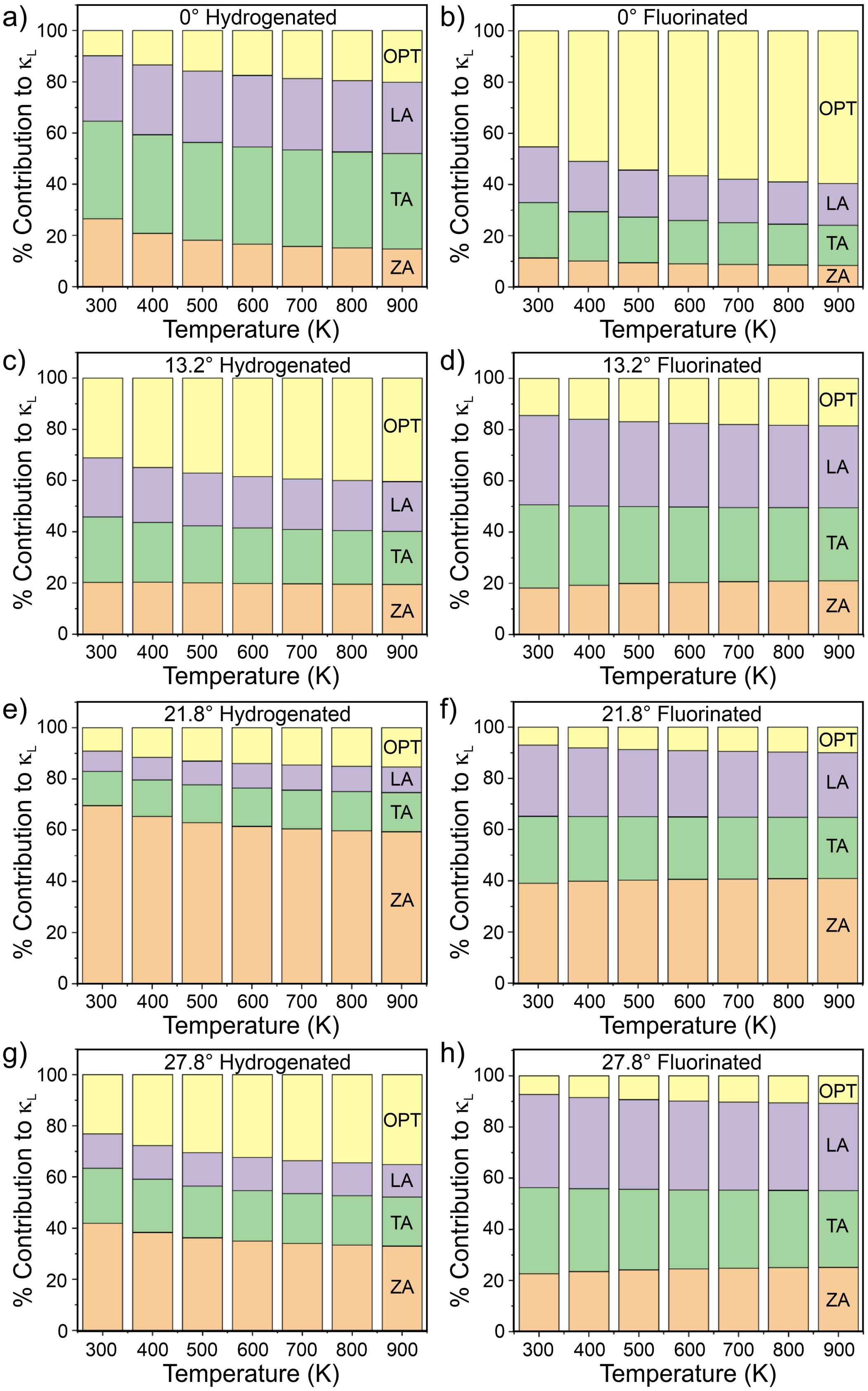

Figure S8. Percentage contribution of different phonon branches to the lattice part of the thermal conductivity of hydrogentated and fluorinated Moiré diamanes with twist angles of a,b) 0°, c,d) 13.2°, e,f) 21.8°, and g,h) 27.8°.
